# Lessons learned through listening to biology students during a transition to online learning in the wake of the COVID‐19 pandemic

**DOI:** 10.1002/ece3.7303

**Published:** 2021-03-13

**Authors:** Eve A. Humphrey, Jason R. Wiles

**Affiliations:** ^1^ Biology Department Lincoln University Lincoln University PA USA; ^2^ Department of Biology Syracuse University Syracuse NY USA

**Keywords:** biology, COVID‐19, distance learning, ecology, evolution, science education

## Abstract

During the Spring Semester of 2020, an outbreak of a novel coronavirus (SARS‐CoV‐2) and the illnesses it caused (COVID‐19) led to widespread cancelling of on‐campus instruction at colleges and universities in the United States and other countries around the world. Response to the pandemic in university settings included a rapid and unexpected shift to online learning for faculty and students. The transition to teaching and learning online posed many challenges, and the experiences of students during this crisis may inform future planning for distance learning experiences during the ongoing pandemic and beyond. Herein, we discuss the experiences of first‐ and second‐year university students enrolled in a biology seminar course as their classes migrated to online environments. Drawing on reported student experiences and prior research and resources, we discuss the ways we will adjust our own teaching for future iterations of the course while offering recommendations for instructors tasked with teaching in online environments.

## Abstract 2

This manuscript is an essay that draws on reported student experiences and prior research and resources during the COVID‐19 pandemic. Responses were used to provide methods or to intrinsically and extrinsically motivating students during their online learning experience as well as ways for educators to assess student needs and make course adjustments.

## 1 INTRODUCTION

In late 2019, an outbreak of pneumonia was reported in Wuhan China and was later tied to a novel strain of human coronavirus. In early 2020, as the novel strain (ultimately named, Coronavirus Disease 2019, or COVID‐19 spread, the World Health Organization (WHO)) provided public health and social measures to slow the spread and reduce the likelihood of future transmission (World Health Organization, 2020b, pp. 72). These measures mandated interpersonal distances of at least one meter, the elimination of mass gatherings, reduction of international travel, and personal quarantine for infected individuals (Cohen & Kupferschmidt, 2020; WHO, 2020b). As a result, universities and colleges were abruptly vacated, and undergraduate students continued their education remotely. Ultimately, researchers and educators in academia were tasked with meeting a variety of unprecedented demands associated with remote teaching and other aspects of distance learning for which, in many cases, they had little to no training or experience (Nicola et al., 2020; Viner et al., 2020). Students as well faced many challenges, including but not limited to variable access to the required technology (Ferdig et al., 2020; Hall et al., 2020; Teräs et al., 2020), frustration with modes of learning they had not intentionally chosen (Aristovnik et al., 2020; Ye et al., 2020), and navigating a host of unexpected and compounding personal and family issues stemming from the pandemic itself (Dhawan, 2020; Hodges et al., 2020; Sahu et al., 2020; Son et al., 2020; but also see Gao et al., 2020; Torales et al., 2020).

Even with such challenges, remote and online learning environments have been an asset to postsecondary institutions during the COVID‐19 pandemic. However, although there is a substantial body of literature on the effectiveness and importance of remote learning (Anderson, 2004; Bell & Federman, 2013; Kim & Bonk, 2006), few institutions or faculty and students expected or were prepared to transition entirely to online settings. As a result, students and faculty faced a variety of challenges as curricular and communicative barriers arose within their new remote and online learning communities. In particular, undergraduate students in biology courses that were once engaged in class lectures, in‐class group interactions, laboratory activities, and field studies were tasked with learning about the natural world from their computer screens in mandated social isolation.

Here, we provide the experiences of undergraduate students in a biology course at a large, private, research‐intensive university (Carnegie R1 designation) in the northeast of the United States during the COVID‐19 pandemic. We also discuss the ways in which student experiences in the Spring of 2020 informed our instruction for future remote courses, and finally, we draw conclusions from our data and pedagogical literature to suggest tools and techniques that may support students engaging in remote learning and provide support for biology educators, particularly in online ecology and evolution courses.

## 2 COURSE DESCRIPTION

We collected student responses under an IRB‐approved protocol (#17‐249). The course in which our participants were enrolled, was titled Introduction to Biology Research, and was based on prior courses of a similar nature implemented by Schmid and Wiles (2019) and Sloane and Wiles (2020). As with previous iterations, this course was designed as a literature‐ and discussion‐based seminar for first‐ and second‐year students majoring in biology or fields related to biology (Table 1). The course occurred in the spring of 2020 and consisted of one section of 15 students. Additionally, all of the students had previously taken one semester of a general biology course that serves as a foundation for all biology majors and related programs at this university. This 2‐credit elective course met once per week, and the first two weeks of instruction centered around different types of scientific writing using two instructor‐assigned primary research articles (Bailey & Coe, 1999; Hossie et al., 2018) and two instructor‐assigned review papers (Lima, 2002; Moloney et al., 2016). Thereafter, students engaged in reading, discussion of, and writing about original research and scholarly reviews around key biological topics on a weekly basis. Students also earned participation credit by completing journal entries that involved a short reflection.

**TABLE 1:** Classification of student majors enrolled in the course

Students' declared majors	Number of students
Biology	5
Biochemistry	2
Forensic Science	2
Neuroscience	2
Biology/Neuroscience	1
Psychology	1
Economics	1
Undeclared	1

During the middle of March, and as COVID‐19 cases continued to increase rapidly in the United States, students in the Introduction to Biological Research course and all other courses were informed that the campus would be closed to in‐person learning. Research for the remainder of the semester and all courses would be continued remotely. The instructor (first author) modified participation requirements for the course and students completed the remaining journal reflections remotely via the existing Web‐based course management system (Blackboard). These assignments were modified to allow the author to capture the experiences of students newly engaged with distance learning, the perceptions of students own learning, and their interactions with faculty during the pandemic. Students responded to these reflection questions (Table 2) two different times during the remote class: the first week of their quarantine at home and then three weeks into their remote learning experience. Questions and student responses were completed through Blackboard, and 14 of the 15 students responded to the questions within the allotted times.

**TABLE 2:** Questions students received through an online format in the first and third weeks of their online experience

Week 1 questions	Week 3 questions
a. How has the pandemic impacted you? How have you responded? b. How do you plan to approach your courses for the rest of the semester? c. Now that your courses will be completely online how do you think this will impact you? Do you prefer online versus in person instruction? d. Is there anything else you would like to mention that you are concerned about?	a. Do you believe professors have adjusted well to teaching online? How or how not, use examples. b. Do you believe your level of learning is similar to being on campus? How or how not? c. What changes/efforts can professors make to improve your learning participation in their courses?

## 3 STUDENT PERCEPTIONS OF REMOTE LEARNING AND CHANGES AS A RESULT OF COVID‐19

In this section, we present some of the students' responses to our questions (Table 2) about their preferences regarding learning environments and their perspectives on remote classes and faculty remote instruction.

### 3.1 Student preferences regarding remote versus face‐to‐face instruction

Overall, almost all of the students (11/14) preferred face‐to‐face instruction compared to the remote format. One student commenting about remote learning stated that they found it “hard to stay engaged and learn the actual content” another student mentioned they preferred, “that connection from an in‐person meeting and [ability] to ask questions promptly.” Two students were in support of remote classes in specific contexts compared to face‐to‐face instruction.

Many students brought up their preference for in‐person laboratory and research classes rather than remote laboratory experiences. One student stated that, “when it [comes] to labs, there isn't a way to make hands on activities the same level as online. I think no matter how you run an online class, being in person will always be better in my opinion.” Also, when it came to remote laboratory courses, several students (5/14 students) highlighted the difficulty in learning on their own. Two students mentioned the desire for video demonstrations of laboratory work and additional structured guidance in their notes when it came to completing remote experiments. Another student expressed greater difficulty with discerning expectations and instructions for projects in a remote setting stating that, “online courses are fine for content‐based lecture classes…but they can be a struggle for project‐based courses just because it's harder to understand what I'm being asked to do.”

### 3.2 Remote course scheduling and motivation

During the first week of remote instruction, the majority of students (10/14 students) emphasized a desire to maintain their typical on‐campus schedule in an effort to have a sense of normalcy and to excel in their courses. Many students commented that they, “[wanted] to stay close to [their] normal weekly routine” or highlighted, “do[ing] classes as normal as possible except sitting at [their] dining room table” as well as, “doing all [their] work during the week so [they] could take advantage of being home with family during the weekends.” Students indicated that they were doing their remote assignments, but also expressed that they had been working merely toward completion, and not necessarily thinking of their online work as “studying” or a need to “memorize” information as they typically would during face‐to‐face instruction because their online exams tended to be open book. There was an emphasis on maintaining a sense of normality and routine as many of their professors opted for asynchronous instruction, rather than expecting students to be present at specific times during the day. However, others, particularly international students who had returned to their home countries, expressed difficulty and concern related to expectations of synchronous class meetings that did not align well to their time zones or other scheduling constraints. Several students (6/14 students) voiced concerns about what they perceived as “constantly changing deadlines” in the courses they were taking and how this impacted their ability to submit assignments at the expected time. Many of the students (12/14) noted that their examinations and assignments transitioned into project‐based assessments and writing assignments with new due dates. Students expressed “fear,” “anxiety,” and “worry” about “missing a deadline because there have been so many emails with each saying something new and different.” Many commented that they did not believe they had the proper organizational and planning skills to maintain their course loads at home. However, students were hopeful that remote courses would mean that their course work was “easier” (a term used by four students) but questioned whether they were actually learning anything (stated by six students).

When we considered student approaches to courses between the first week of quarantine compared to the third week, we found that their self‐reported level of motivation (a perceived factor in students' remote experiences) differed. Motivation is an internal reason or state that influences and sustains human behavior and a student's motivation to learn (Brophy, 1999). Many students commented on their lack of motivation or difficulty focusing and maintaining the schedules they previously set in week one. Specifically, students commented that they “had less motivation to learn since all the exams [were] open book,” and that location and constant presence of family members diminished their ability to focus or find a place to study. Eight of the 15 students highlighted the benefit of libraries and coffee shops as their main study areas when in‐person instruction was available. Those eight students emphasized that those study locations allowed them to focus on their materials; however, motivation and focus decreased when they worked from home and lost access to those areas. Several students (7/15) reported difficulties in finding a space dedicated to work and focus, away from family. Two students, who had both listed initial goals for their remote learning as developing a good schedule and “finishing the semester with a bang,” only two weeks later characterized their goal for finishing the semester as “just getting by.”

### 3.3 Student perceptions of course effectiveness & their own learning

Several of our student participants did not believe that their remote courses were as effective as compared to the face‐to‐face instruction that occurred earlier in the semester. One student, when asked if they felt their level of learning was similar to their on campus classes (Table 2, Week 3), answered that they did not believe they were learning as much through video lectures, particularly because “professors are posting, but I am unable to really ask questions.” The student even considered how changes would impact their future course selections by saying, “it is concerning to me as a biochemistry major because all the classes I am taking next semester require my previous knowledge.” Another student stated that they disliked the remote classes because it, “[was] harder to stay engaged and actually learn the content.”

We did find, however, that students in this course judged their ability to learn science content not only on the context of the course and the materials they were given, but also the course's influence on their emotional state. One student highlighted this when saying, “I haven't learned material as effectively, but, it has significantly reduced my stress levels.” A few students (3/14) also mentioned how difficulties with time zones, Internet connection, and the remote learning system hindered some of their learning process.

### 3.4 Student perception of faculty during remote instruction

During this unprecedented time, faculty made quick and often substantial changes to their courses for remote instruction. As a result, students were faced with the task of realigning their expectations as faculty provided different curricula and assessments for the semester. We found that overall in this case study, students used positive and supportive language about their professors' efforts to transition courses remotely. The majority of our students (12/14) believed their professors “adjusted well” (a term used by 6 of the students) and these students only provided specific examples when professors were less accommodating.

When responding about their professors' adjustments and transition to remote learning, all of the students made reference to and took into account the timing of the pandemic mentioning the “short notice” and “drastic changes” as a factor they considered in analyzing their professor's engagement and remote teaching. Students' responses included examples of video recordings, voiceovers of PowerPoint slides, increased time for assignment due dates, and open‐book quizzes and examinations as methods of adequate support from faculty during the transition to remote learning. Students voiced concerns with synchronous teaching and online issues, or, having difficulty connecting to remote courses at specific times in the morning due to high user volume. Some students found that more than one of their classes were live at the same time and had to choose between which class to participate in synchronously, which was particularly challenging as some professors did not record and upload their lectures. One student noted, “other teachers have a bit of difficult time though it is not their fault, they are trying to procure ways to help. Some class times are cut in order to help students [with] internet [challenges].” Only two of the fourteen students believed that their professors had not adjusted well to remote teaching. One student stated that, “I do not believe the professors have adjusted very well to remote teaching. Especially since teachers speed through examples and teaching in order to keep a small class time.” The other students provided similar comments when they noted,

I do not believe my professors have adjusted well to teaching remotely. I also do not believe it is their fault, some of my classes with lots of students and assignments have taken a huge hit. There has not been a lot of changes in structure which make it hard to comprehend the information.

## 4 USING STUDENT RESPONSES DURING COVID‐19 TO INFORM COURSE DESIGN & TEACHING

We used student feedback about their transitions to remote learning in the Spring of 2020 to inform our future courses and to consider how to improve the online learning experience of students. We understand that there are several limitations to the study: A small sample size makes it difficult to extrapolate student experience to larger biology courses or to make generalizable statements about undergraduate biology students' perceptions of remote learning. Additionally, student responses in such a small context, although elucidating, may limit inferences about online learning and best practices to specific courses. We do believe that students were candid with their statements, reliability of these qualitative data would be bolstered by multiple iterations of the course and if collection of student responses were taken from a variety of biology courses of various sizes and topics. Notably, student responses in our course reflected similar student responses in studies (of varying sizes) on perceived support (Lee et al., 2011) particularly centered around clear course expectations (Palmer & Holt, 2009) and motivation and lack of community (Song et al., 2004) in online courses. Ultimately, we used our student responses and other online student perception studies (Care et al., 2001; Deci et al., 1991; Schilling & Schilling, 1999; Selim, 2007; Smart & Cappel, 2006) to revise the course for online delivery in the future and leaned heavily on best practices to support student engagement and learning online.

In the first week of the quarantine, students were apprehensive about the changes to their learning environment and highlighted community as an important aspect of their learning. In the first week, students were focused on developing schedules that reflected their on‐campus routines. However, in the three‐week check‐in, several students expressed difficulty in maintaining schedules and motivation, and many of them expressed frustration with perceived inflexibility on the part of some of their instructors. The majority of the students in this case study reported difficulties creating and maintaining work and study schedules that supported their learning. As a result, this future online course will begin with a discussion with students to explicitly discuss the potential for this global health crisis to influence the productivity and mental health of all those involved (Gibbons et al., 2011; Son et al., 2020). At the onset of the course we will offer university resources, data and peer reviewed materials that focus on organizational and coping skills in order to support student engagement and completion of assignments (Pfefferbaum & North, 2020; Sitler, 2009). Our initial contact with students will include transparency about course expectations (Felten & Finley, 2019; Wisehart, 2004) and a clear schedule of due dates and details of the remote or online course before any assignments or lectures are posted.

Previous research reflects our own student responses, and both highlight the importance of support from the instructor for distance learners, specifically with an emphasis on *how* the instructor sets up the online course (Care et al., 2001; Selim, 2007). Additionally, evidence from a metanalysis surveying students at 29 universities indicated that students prefer three important fields of expertise from their online instructor: “(a) structure and coherence of the learning material and course, (b) stimulation of learning motivation, and (cc) facilitation of collaborative learning structures of the learning content” (Paechter et al., 2010). Findings from this metanalysis address some of the difficulties our students faced, particularly in regard to creating coherent learning material and with supporting students' motivation during their online learning. Therefore, we have considered ways to (a) intrinsically and extrinsically motivate students, and (b) check in with students throughout the course (but critically within the first few weeks) to assess student learning and perceptions and we provide examples of these efforts for our future course below.

Motivation was a common factor in our student responses and earlier studies highlight that motivation depends on students' “tendency to find academic activities meaningful and worthwhile and to derive the intended academic benefits from them” (Brophy, 1999, pp.205–206; Glynn et al., 2005). Drawing from student responses and prior research and resources that reflect similar needs of our students (Childers & Berner, 2000; Covington, 2000; Deci et al., 1991; Linnenbrink & Pintrich, 2000; Schilling & Schilling, 1999), we will use the following suggestions and strategies for maintaining student motivation online specifically for biology and ecology and evolution educators:

1. Clearly communicate and maintain expectations at the beginning of the semester and throughout the course. This first approach can be used for biology classes as small as 20 students or as large as 200 students. Expectations of student learning outcomes or goals will be explicit and clear for students to connect to the work that they do. We will provide student learning outcomes in the syllabus and reiterate goals during introduction of the course and throughout the semester. Future courses will include explicit alignment of assessments with the introduced learning outcomes, and students will be asked to reflect on how their work and participation matched learning goals at the end of each activity or assessment (example of learning goals in S.1). These learning outcomes are created at the discretion of the educator, but other institutions may require separate university and departmental learning outcomes to align with student learning goals. In a traditional biological course, there is opportunity for experimentation and model testing that is developed by the students. Traditional biological classrooms centered around learning through exploration and experimentation allow students to work with their instructors to develop learning goals. However, online instruction may require students to have more explicit communication about learning outcomes and expectations, and they may need those expectations to be repeated, in multiple modes (e.g., the course syllabus, emails, online announcements, in recorded lectures, and associated with the assignments themselves) throughout the course.
2. Provide students with the opportunity to make more decisions on online content. Online settings often necessitate reading of review papers or participation in discussion boards. Students are often motivated when the educator allows students to explore the ecological and evolutionary topics that are most interesting to them and often take students out of typically laboratory setting (Barnett et al., 2011; Braund & Reiss, 2006; Spronken‐Smith et al., 2011). In order to extrinsically motivate students, we will also offer examples across taxa or environments related to particular evolutionary concepts from which students can select. We will also provide students with the opportunity to find videos online that align with our content. Students can upload videos through online learning platform message boards. Alternately, students can upload videos via Google docs or a mobile video discussion platform called Flipgrid (https://info.flipgrid.com). For example, a future week of remote teaching will likely involve discussing the research of a faculty member who studies mate guarding, using our online learning platform or Flipgrid, we may introduce the topic before reading the faculties research by asking students to search for videos of mate guarding or other animal behaviors to share with the class and discuss the behaviors in the video. The examples we mention above are useful in smaller classes, preferably smaller than 30 students, as instructor–student interaction are important for each decision making and video example. Alternatively (and for larger courses), we will also give students a variety of ecologically relevant case studies (Table 3) that align with faculty research and allow students to select one of personal interest for their assignment. This may give students some control on the tasks they use to learn about weekly content and provide a variety of topics and student perspectives for discussion. Educators may be hesitant to provide a variety of case studies to students when considering trade‐offs between student motivation and time spent grading tasks. However, many case study resources (Table 3) offer case studies and corresponding keys/solutions for educators to reduce grading load.
3. We also suggest, and will, implement course activities that increase the cognitive demand of tasks and provide students with challenges that are at the edges of, or even marginally beyond, their educational level (Tekkumru‐Kisa & Stein, 2015; Tekkumru‐Kisa et al., 2015). We will provide discussion questions and in class tasks (research article summaries, experimental design assessment, statistical analyses) that are more aligned with those that ecologists and evolutionary biologists must grapple with daily. These discussion questions and tasks can be applied to any class size given the content of the lesson and learning outcomes of the students. For example, throughout the semester we will use evidence based on graphs and data tables from published research papers. Student activities will center around using the graph reading skills they develop in the course to develop conclusions based on tasks that involve graphs that coincide with data tables. This will provide students the opportunity to feel more engaged with the work, it will align with class discussions and also give us as educators the opportunity to assess critical thinking skills. This method may also give students the opportunity to understand how scientists interpret data and understand that the “doing” of science can occur at one's computer and not only in the field or laboratory. Additionally, case studies (Table 3) that encourage students to utilize the content they are learning online with the practices that ecologists and evolutionary biologists use (statistics in R, writing results, citing previous research) offer a diversity of subject matter and motivating challenge to student learning (Bonney, 2015).
4. Give students the opportunity to reflect on what they have learned and make connections with their own world and goals through writing and online discussion. Incorporating an online discussion board or journaling can give students the opportunity to process what they are learning, but these techniques also allow educators to identify misconceptions and gaps in students' understandings (Callis‐Duehl et al., 2018; Halim et al., 2018; Williams et al., 2016). By asking simple questions like, “How does this new information impact the way you view population growth?” or “What trophic interactions have you seen in your backyard or around where you live?” students may begin to make connections between what they are learning and their own experiences that generate perceptions of practical relevance. These reelections can occur in the form of written responses (for smaller classes) or via online polling and presentation platforms (Kahoot © ‐https://kahoot.com Zoom‐ https://zoom.us.com, Poll Everywhere https://www.polleverywhere.com) for larger classes.

**TABLE 3:** Examples of activities and assessments that can be modified for remote learning by ecology and evolution educators

Assessment/Activity Type	Description	Data received	Implementation/examples
Quick course diagnostic (Millis & Vasquez, 2010)	Diagnostic tool that utilizes pre and post student interviews.	Satisfaction with course Qualitative data on course satisfaction Course strengths &weaknesses Learning outcomes met Data multiple times during the course Individual & group data Pre and post assessment	Can stimulate online questionnaire Students indicate course satisfaction with a number (1–5) Students provide 1–2‐word descriptor of course Students look at list of student learning outcomes and select which were achieved Students discuss strength & weaknesses of course in groups
Minute Paper (Angelo & Cross, 1993; Chizmar & Ostrosky, 1998; Stead, 2005; Vonderwell, 2004)	Metacognitive tool to get feedback on specific areas of confusion or difficulty at the end of a lesson	Qualitative data Clarity of lecture or activity Student links between learning outcomes and class tasks Comparison of student understanding between/across classes within a course	Students given about 60 s (or class appropriate amount of time) What was confusing today in class? What was the most important thing you learned today? How does today's topic connect with what we discussed last class?
Case Studies (Feagin et al., 1991; Herreid, 1994; Kobori et al., 2016; Millis & Cottell, 1997; Popil, 2011; Taber, 2000)	An active learning tool that engages students in learning through inquiry. Also supports students' learning as they explore ecological and evolutionary phenomena.	Educators assess student responses or assign students' peers to read and provide feedback to others' responses to case study assignments Quick assessment of students' incorporation of course information with data Assess students' ability to understand statistics, summarize data, discuss methodology	Over 800 peer reviewed case studies at the National Center for Case Study Teaching in Science (https://sciencecases.lib.buffalo.edu) Students become researchers as they work through questions and data to come to conclusions about ecological and evolutionary phenomena Can be given after discussing a specific topic to give real word application to a lecture or concept
Muddiest Point (Carberry et al., 2013; Krause et al., 2014; Mosteller, 1989)	An activity that provides students the opportunity to reflect and communicate what was confusing or “muddy” during a class session.	Educators receive formative feedback from students Understanding of clarity of lecture or review points Identifying required knowledge that is missing or misunderstood in students Data collected during multiple classes across the course	Using clicker questions to receive quick data what percent of the class found difficult with specific lecture topics or Students can “traffic light” topics, and write most difficult topic highlighted in red, somewhat difficult topic highlighted in yellow and topics completely understood highlighted in green Used in a discussion board where students can “like” or agree on “muddiest point” during online lecture Use collected data of “muddiest point” as an examination review tool
Citizen Science (Kobori et al., 2016; Silvertown, 2009)		Quick assessment of students' incorporation of course information with data Determine student's perspective on the efficacy of citizen research Assess students' ability to understand statistics, summarize data, discuss methodology	e.g., citizenscience.gov, science.nasa.gov, zooniverse.org, ecologyproject.org, etc. Educators can also create class data for students to practice statistics, summarize data, discuss methodology and explore the efficacy of citizen science itself

Although students may have deadlines and develop their own expectations about their approach to the course, online formats provide a variety of motivational, and situational difficulties that students may or may not be aware of. If faculty take stock of their courses and assess students' needs within the first few weeks of an online course, they can provide scaffolding or additional support to students and make appropriate adjustments to the course before unforeseen problems arise. Using student responses, we have identified and propose five different online and adaptable activities (Table 3) for educators to use. We will personally incorporate four of the five activities (Quick Course Diagnostics, Minute Paper, Case Studies, Muddiest Point) in the upcoming semester to support our students during this online synchronized course. These activities can serve as an assessment to student learning and provide opportunity for intentional engagement with students throughout the semester.

## 5 CONCLUSION

The shift to remote teaching and learning created a variety of challenges and experiences for students in the Spring of 2020, and instructors had to adjust their content and curriculum for student learning and engagement accordingly. Here, we provided ways in which we will continue to adjust our own courses to intrinsically and extrinsically motivate students during their online learning experience as well as ways for educators to assess student needs and make course adjustments. Although these challenges arose in a unique context, which will hopefully not last, we were pleased to find that our students were flexible and attempted to meet the new changes head on in the midst of stay‐at‐home orders. Identifying student perceptions in this unique context has given us the opportunity to re‐address and develop new techniques that will continue to support the education of our students. This work also serves as a reminder of the resilience of students and an encouraging glimpse into the future researchers and educators we will see in the field of ecology and evolution.

## CONFLICT OF INTEREST

The authors state that they have no conflicting interests.

## AUTHOR CONTRIBUTION

**Eve Humphrey:** Conceptualization (lead); Data curation (lead); Formal analysis (lead); Methodology (lead); Project administration (lead); Writing‐original draft (lead). **Jason R Wiles:** Supervision (lead); Writing‐review & editing (supporting).

## Supporting information

Supplementary Material

## References

[ece37303-bib-0001] Anderson, T. (2004). Towards a theory of online learning. Theory and Practice of Online Learning, 2, 109–119.

[ece37303-bib-0002] Angelo, T. A. , & Cross, K. P. (1993). Classroom assessment techniques: A handbook for college teachers (pp. 148–153). Minute paper.

[ece37303-bib-0003] Aristovnik, A. , Keržič, D. , Ravšelj, D. , Tomaževič, N. , & Umek, L. (2020). Impacts of the COVID‐19 pandemic on life of higher education students: A global perspective. 10.1016/j.dib.2021.107659 PMC8634691 34869802

[ece37303-bib-0004] Bailey, M. T. , & Coe, C. L. (1999). Maternal separation disrupts the integrity of the intestinal microflora in infant rhesus monkeys. Developmental Psychobiology: The Journal of the International Society for Developmental Psychobiology, 35(2), 146–155. 10.1002/(SICI)1098-2302(199909)35:2<146:AID-DEV7>3.0.CO;2-G 10461128

[ece37303-bib-0005] Barnett, M. , Vaughn, M. H. , Strauss, E. , & Cotter, L. (2011). Urban environmental education: Leveraging technology and ecology to engage students in studying the environment. International Research in Geographical and Environmental Education, 20(3), 199–214. 10.1080/10382046.2011.588501

[ece37303-bib-0006] Bell, B. S. , & Federman, J. E. (2013). E‐learning in postsecondary education. The Future of Children, 165–185. 10.1353/foc.2013.0007 25522650

[ece37303-bib-0007] Bonney, K. M. (2015). Case study teaching method improves student performance and perceptions of learning gains. Journal of Microbiology & Biology Education, 16(1), 21. 10.1128/jmbe.v16i1.846 25949753 PMC4416499

[ece37303-bib-0008] Braund, M. , & Reiss, M. (2006). Towards a more authentic science curriculum: The contribution of out‐of‐school learning. International Journal of Science Education, 28(12), 1373–1388. 10.1080/09500690500498419

[ece37303-bib-0009] Brophy, J. (1999). Toward a model of the value aspects of motivation in education: Developing appreciation for. Educational Psychologist, 34(2), 75–85. 10.1207/s15326985ep3402_1

[ece37303-bib-0010] Callis‐Duehl, K. , Idsardi, R. , Humphrey, E. A. , & Gougis, R. D. (2018). Missed opportunities for science learning: Unacknowledged unscientific arguments in asynchronous online and face‐to‐face discussions. Journal of Science Education and Technology, 27(1), 86–98. 10.1007/s10956-017-9710-4

[ece37303-bib-0011] Carberry, A. , Krause, S. , Ankeny, C. , & Waters, C. (2013). “Unmuddying” course content using muddiest point reflections. IEEE (pp. 937–942). IEEE Frontiers in Education Conference (FIE ). 10.1109/FIE.2013.6684966

[ece37303-bib-0012] Care, W. D. , & Scanlan, J. M. (2001). Planning and managing the development of courses for distance delivery: Results from qualitative study. Online Journal of Distance Learning Administration, 4(2).

[ece37303-bib-0013] Childers, J. L. , & Berner, R. T. (2000). General education issues, distance education practices: Building community and classroom interaction through the integration of curriculum, instructional design, and technology. The Journal of General Education, 49(1), 53–65. 10.1353/jge.2000.0001

[ece37303-bib-0014] Chizmar, J. F. , & Ostrosky, A. L. (1998). The one‐minute paper: Some empirical findings. The Journal of Economic Education, 29(1), 3–10. 10.1080/00220489809596436

[ece37303-bib-0015] Cohen, J. , & Kupferschmidt, K. (2020). Countries test tactics in 'war' against COVID‐19. Science, 367(6484), 1287–1288. 32193299 10.1126/science.367.6484.1287

[ece37303-bib-0016] Covington, M. V. (2000). Goal theory, motivation, and school achievement: An integrative review. Annual Review of Psychology, 51(1), 171–200. 10.1146/annurev.psych.51.1.171 10751969

[ece37303-bib-0017] Deci, E. L. , Vallerand, R. J. , Pelletier, L. G. , & Ryan, R. M. (1991). Motivation and education: The self‐determination perspective. Educational Psychologist, 26(3–4), 325–346.

[ece37303-bib-0018] Dhawan, S. (2020). Online learning: A panacea in the time of COVID‐19 crisis. Journal of Educational Technology Systems, 49(1), 5–22. 10.1177/0047239520934018

[ece37303-bib-0019] J. R. Feagin , A. M. Orum , & G. Sjoberg . (Eds.) (1991). A case for the case study. UNC Press Books.

[ece37303-bib-0020] Felten, P. , & Finley, A. (2019). Transparent design in higher education teaching and leadership: A guide to implementing the transparency framework institution‐wide to improve learning and retention. Stylus Publishing LLC.

[ece37303-bib-0021] Ferdig, R. E. , Baumgartner, E. , Hartshorne, R. , Kaplan‐Rakowski, R. , & Mouza, C. (2020). Teaching, technology, and teacher education during the covid‐19 pandemic: Stories from the field. Association for the Advancement of Computing in Education (AACE).

[ece37303-bib-0022] Gao, J. , Zheng, P. , Jia, Y. , Chen, H. , Mao, Y. , Chen, S. , Wang, Y. I. , Fu, H. , & Dai, J. (2020). Mental health problems and social media exposure during COVID‐19 outbreak. PLoS One, 15(4), e0231924. 10.2139/ssrn.3541120 32298385 PMC7162477

[ece37303-bib-0023] Gibbons, C. , Dempster, M. , & Moutray, M. (2011). Stress, coping and satisfaction in nursing students. Journal of Advanced Nursing, 67(3), 621–632. 10.1111/j.1365-2648.2010.05495.x 21077931

[ece37303-bib-0024] Glynn, S. M. , Aultman, L. P. , & Owens, A. M. (2005). Motivation to learn in general education programs. The Journal of General Education, 150–170. 10.1353/jge.2005.0021

[ece37303-bib-0025] Halim, A. S. , Finkenstaedt‐Quinn, S. A. , Olsen, L. J. , Gere, A. R. , & Shultz, G. V. (2018). Identifying and remediating student misconceptions in introductory biology via writing‐to‐learn assignments and peer review. Cbe—life Sciences Education, 17(2), ar28. 10.1187/cbe.17-10-0212 29749850 PMC5998326

[ece37303-bib-0026] Hall, T. , Connolly, C. , Ó Grádaigh, S. , Burden, K. , Kearney, M. , Schuck, S. , Bottema, J. , Cazemier, G. , Hustinx, W. , Evens, M. , Koenraad, T. , Makridou, E. , & Kosmas, P. (2020). Education in precarious times: A comparative study across six countries to identify design priorities for mobile learning in a pandemic. Information and Learning Sciences.

[ece37303-bib-0027] Herreid, C. F. (1994). Case studies in science–a novel method of science education. Journal of College Science Teaching, 23(4), 221–229.

[ece37303-bib-0028] Hodges, C. , Moore, S. , Lockee, B. , Trust, T. , & Bond, A. (2020). The difference between emergency remote teaching and online learning. Educause Review, 27.

[ece37303-bib-0029] Hossie, T. J. , MacFarlane, S. , Clement, A. , & Murray, D. L. (2018). Threat of predation alters aggressive interactions among spotted salamander (Ambystoma maculatum) larvae. Ecology and Evolution, 8(6), 3131–3138. 29607012 10.1002/ece3.3892 PMC5869354

[ece37303-bib-0030] Kim, K. J. , & Bonk, C. J. (2006). The future of online teaching and learning in higher education. Educause Quarterly, 29(4), 22–30.

[ece37303-bib-0031] Kobori, H. , Dickinson, J. L. , Washitani, I. , Sakurai, R. , Amano, T. , Komatsu, N. , Kitamura, W. , Takagawa, S. , Koyama, K. , Ogawara, T. , & Miller‐Rushing, A. J. (2016). Citizen science: A new approach to advance ecology, education, and conservation. Ecological Research, 31(1), 1–19. 10.1007/s11284-015-1314-y

[ece37303-bib-0032] Krause, S. , Baker, D. R. , Carberry, A. , Alford, T. , Ankeny, J. , Koretsky, M. , & Chan, C. (2014). Characterizing and addressing student learning issues and misconceptions (SLIMs) in materials science with muddiest point reflections and fast formative feedback. In 121st ASEE annual conference and exposition: 360 degrees of engineering education (pp. 937–942). American Society for Engineering Education. 10.18260/1-2--20164

[ece37303-bib-0033] Lee, S. J. , Srinivasan, S. , Trail, T. , Lewis, D. , & Lopez, S. (2011). Examining the relationship among student perception of support, course satisfaction, and learning outcomes in online learning. The Internet and Higher Education, 14(3), 158–163. 10.1016/j.iheduc.2011.04.001

[ece37303-bib-0034] Lima, S. L. (2002). Putting predators back into behavioral predator–prey interactions. Trends in Ecology & Evolution, 17(2), 70–75. 10.1016/S0169-5347(01)02393-X

[ece37303-bib-0035] Linnenbrink, E. A. , & Pintrich, P. R. (2000). Multiple pathways to learning and achievement: The role of goal orientation in fostering adaptive motivation, affect, and cognition. Intrinsic and extrinsic motivation (pp. 195–227). Academic Press.

[ece37303-bib-0036] Millis, B. J. , & Cottell, P. G. Jr (1997). Cooperative learning for higher education faculty. Series on higher education (pp. 85067–93889). Oryx Press.

[ece37303-bib-0037] Millis, B. J. , & Vazquez, J. (2010). Essays on teaching excellence. Toward the Best in the Academy. the Professional & Organizational Development Network. Higher Education, 22(4).

[ece37303-bib-0038] Moloney, R. D. , Johnson, A. C. , O'mahony, S. M. , Dinan, T. G. , Greenwood‐Van Meerveld, B. & Cryan, J. F. (2016). Stress and the microbiota–gut–brain axis in visceral pain: Relevance to irritable bowel syndrome. CNS Neuroscience & Therapeutics, 22(2), 102–117. 26662472 10.1111/cns.12490 PMC6492884

[ece37303-bib-0039] Mosteller, F. (1989). The 'Muddiest point in the lecture' as a feedback device. On Teaching and Learning: The Journal of the Harvard‐Danforth Center, 3, 10–21.

[ece37303-bib-0040] Nicola, M. , Alsafi, Z. , Sohrabi, C. , Kerwan, A. , Al‐Jabir, A. , Iosifidis, C. , Agha, M. , & Agha, R. (2020). The socio‐economic implications of the coronavirus pandemic (COVID‐19): A review. International Journal of Surgery (London, England), 78, 185. 10.1016/j.ijsu.2020.04.018 PMC7162753 32305533

[ece37303-bib-0041] Paechter, M. , Maier, B. , & Macher, D. (2010). Students' expectations of, and experiences in e‐learning: Their relation to learning achievements and course satisfaction. Computers & Education, 54(1), 222–229. 10.1016/j.compedu.2009.08.005

[ece37303-bib-0042] Palmer, S. R. , & Holt, D. M. (2009). Examining student satisfaction with wholly online learning. Journal of Computer Assisted Learning, 25(2), 101–113. 10.1111/j.1365-2729.2008.00294.x

[ece37303-bib-0043] Pfefferbaum, B. , & North, C. S. (2020). Mental health and the Covid‐19 pandemic. New England Journal of Medicine. 10.1056/NEJMp2008017 32283003

[ece37303-bib-0044] Popil, I. (2011). Promotion of critical thinking by using case studies as teaching method. Nurse Education Today, 31(2), 204–207. 10.1016/j.nedt.2010.06.002 20655632

[ece37303-bib-0045] Sahu, P. (2020). Closure of universities due to Coronavirus Disease 2019 (COVID‐19): Impact on education and mental health of students and academic staff. Cureus, 12(4), e7541. 32377489 10.7759/cureus.7541 PMC7198094

[ece37303-bib-0046] Schilling, K. M. , & Schilling, K. L. (1999). Increasing expectations for student effort. About Campus, 4(2), 4–10.

[ece37303-bib-0047] Schmid, K. M. , & Wiles, J. R. (2019). An introduction to biological research course for undergraduate biology students. Journal of College Science Teaching, 49(1).

[ece37303-bib-0048] Selim, H. M. (2007). Critical success factors for e‐learning acceptance: Confirmatory factor models. Computers & Education, 49(2), 396–413. 10.1016/j.compedu.2005.09.004

[ece37303-bib-0049] Silvertown, J. (2009). A new dawn for citizen science. Trends in Ecology & Evolution, 24(9), 467–471. 10.1016/j.tree.2009.03.017 19586682

[ece37303-bib-0050] Sitler, H. C. (2009). Teaching with awareness: The hidden effects of trauma on learning. The Clearing House: A Journal of Educational Strategies, Issues and Ideas, 82(3), 119–124. 10.3200/TCHS.82.3.119-124

[ece37303-bib-0051] Sloane, J. D. , & Wiles, J. R. (2020). Communicating the consensus on climate change to college biology majors: The importance of preaching to the choir. Ecology and Evolution, 10(2), 594–601. 10.1002/ece3.5960 32015828 PMC6988523

[ece37303-bib-0052] Smart, K. L. , & Cappel, J. J. (2006). Students' perceptions of online learning: A comparative study. Journal of Information Technology Education: Research, 5(1), 201–219. 10.2139/ssrn.3524610

[ece37303-bib-0053] Son, C. , Hegde, S. , Smith, A. , Wang, X. , & Sasangohar, F. (2020). Effects of COVID‐19 on college students' mental health in the United States: Interview survey study. Journal of Medical Internet Research, 22(9), e21279. 10.2196/21279 32805704 PMC7473764

[ece37303-bib-0054] Song, L. , Singleton, E. S. , Hill, J. R. , & Koh, M. H. (2004). Improving online learning: Student perceptions of useful and challenging characteristics. The Internet and Higher Education, 7(1), 59–70. 10.1016/j.iheduc.2003.11.003

[ece37303-bib-0055] Spronken‐Smith, R. A. , Walker, R. , Dickinson, K. J. M. , Closs, G. P. , Lord, J. M. , & Harland, T. (2011). Redesigning a curriculum for inquiry: An ecology case study. Instructional Science, 39(5), 721–735. 10.1007/s11251-010-9150-5

[ece37303-bib-0056] Stead, D. R. (2005). A review of the one‐minute paper. Active Learning in Higher Education, 6(2), 118–131. 10.1177/1469787405054237

[ece37303-bib-0057] Taber, K. S. (2000). Case studies and generalizability: Grounded theory and research in science education. International Journal of Science Education, 22(5), 469–487. 10.1080/095006900289732

[ece37303-bib-0058] Tekkumru Kisa, M. , & Stein, M. K. (2015). Learning to see teaching in new ways: A foundation for maintaining cognitive demand. American Educational Research Journal, 52(1), 105–136.

[ece37303-bib-0059] Tekkumru‐Kisa, M. , Stein, M. K. , & Schunn, C. (2015). A framework for analyzing cognitive demand and content‐practices integration: Task analysis guide in science. Journal of Research in Science Teaching, 52(5), 659–685. 10.1002/tea.21208

[ece37303-bib-0060] Teräs, M. , Suoranta, J. , Teräs, H. , & Curcher, M. (2020). Post‐Covid‐19 education and education technology 'solutionism': A seller's market. Postdigital Science and Education, 2(3), 863–878. 10.1007/s42438-020-00164-x PMC7355515 40477107

[ece37303-bib-0061] Torales, J. , O'Higgins, M. , Castaldelli‐Maia, J. M. , & Ventriglio, A. (2020). The outbreak of COVID‐19 coronavirus and its impact on global mental health. International Journal of Social Psychiatry, 66(4), 317–320. 10.1177/0020764020915212 32233719

[ece37303-bib-0062] Viner, R. M. , Russell, S. J. , Croker, H. , Packer, J. , Ward, J. , Stansfield, C. , Mytton, O. , Bonell, C. , & Booy, R. (2020). School closure and management practices during coronavirus outbreaks including COVID‐19: A rapid systematic review. The Lancet Child & Adolescent Health, 4(5), 397–404. 10.1016/S2352-4642(20)30095-X 32272089 PMC7270629

[ece37303-bib-0063] Vonderwell, S. (2004). Assessing online learning and teaching: Adapting the minute paper. TechTrends, 48(4), 29–31. 10.1007/BF02763442

[ece37303-bib-0064] Williams, J. J. , Lombrozo, T. , Hsu, A. , Huber, B. , & Kim, J. (2016). Revising learner misconceptions without feedback: Prompting for reflection on anomalies. In Proceedings of the 2016 CHI conference on human factors in computing systems (pp. 470–474). New York, NY, United States: Association for Computing Machinery.

[ece37303-bib-0065] Wisehart, R. (2004). Nurturing passionate teachers: Making our work transparent. Teacher Education Quarterly, 31(4), 45–53.

[ece37303-bib-0066] World Health Organization . (2020b). Coronavirus disease 2019 (COVID‐19): situation report. 72.

[ece37303-bib-0067] Ye, Z. , Yang, X. , Zeng, C. , Wang, Y. , Shen, Z. , Li, X. , & Lin, D. (2020). Resilience, social support, and coping as mediators between covid‐19‐related stressful experiences and acute stress disorder among college Students in China. Applied Psychology: Health and Well‐being, 15, 10.1111/aphw.12211 PMC7405224 32666713

