# Supplementary material for: Lessons learned through listening to biology students during a transition to online learning in the wake of the COVID‐19 pandemic
Source: Ecol Evol. 2021 Mar 13;11(8):3450–8. doi: 10.1002/ece3.7303 (PMC8057322; doi:10.1002/ece3.7303)
Supplement: Supplementary file 1 — Supplementary Material [file ECE3-11--s001.docx]

**SUPPLEMENTAL MATERIAL**

S.1. Example of Couse Learning Outcomes for an undergraduate Introduction to Biological Research Course

| Learning Outcome  Students will be able to: | Assessment |
| --- | --- |
| 1. Read and summarize important scientific questions, methods and results of primary research articles, | Paper summaries (graded via rubric), Presentation of research lab (background on research, laboratory members, grants funding research etc.) |
| 1. Develop questions and participate in discussion about the culture, research questions and methods in laboratories at their university and about research in general | Ability to discuss confounding factors of research; participation in class discussion, argumentation about specific point; number of times participated |
| 1. Identify the types of biological research in which the students would like to participate during their time as undergraduates at the university | A list of three potential research labs; Interview or meeting with potential research lab Principal Investigator; acceptance into laboratory for following semester; addition to wait list for research opportunity |
